# MJ-GAN: Generative Adversarial Network with Multi-Grained Feature Extraction and Joint Attention Fusion for Infrared and Visible Image Fusion

**DOI:** 10.3390/s23146322

**Published:** 2023-07-12

**Authors:** Danqing Yang, Xiaorui Wang, Naibo Zhu, Shuang Li, Na Hou

**Affiliations:** 1 School of Optoelectronic Engineering, Xidian University, Xi’an 710071, China; 1922550996@qq.com; 2 Research Institute of System Engineering, PLA Academy of Military Science, Beijing 100091, China; 1252592679@qq.com (N.Z.); 834588643@qq.com (S.L.); xiangku860101@163.com (N.H.)

**Keywords:** infrared and visible image fusion (IVIF), multi-scale feature extraction (MFE), joint attention fusion (JAF), generative adversarial network (GAN), self-attention mechanism (SAM)

## Abstract

The challenging issues in infrared and visible image fusion (IVIF) are extracting and fusing as much useful information as possible contained in the source images, namely, the rich textures in visible images and the significant contrast in infrared images. Existing fusion methods cannot address this problem well due to the handcrafted fusion operations and the extraction of features only from a single scale. In this work, we solve the problems of insufficient information extraction and fusion from another perspective to overcome the difficulties in lacking textures and unhighlighted targets in fused images. We propose a multi-scale feature extraction (MFE) and joint attention fusion (JAF) based end-to-end method using a generative adversarial network (MJ-GAN) framework for the aim of IVIF. The MFE modules are embedded in the two-stream structure-based generator in a densely connected manner to comprehensively extract multi-grained deep features from the source image pairs and reuse them during reconstruction. Moreover, an improved self-attention structure is introduced into the MFEs to enhance the pertinence among multi-grained features. The merging procedure for salient and important features is conducted via the JAF network in a feature recalibration manner, which also produces the fused image in a reasonable manner. Eventually, we can reconstruct a primary fused image with the major infrared radiometric information and a small amount of visible texture information via a single decoder network. The dual discriminator with strong discriminative power can add more texture and contrast information to the final fused image. Extensive experiments on four publicly available datasets show that the proposed method ultimately achieves phenomenal performance in both visual quality and quantitative assessment compared with nine leading algorithms.

## 1. Introduction

Multimodality image fusion is the synthesis of multiple original images of objects in the same scene captured simultaneously from different sensors into a single new image that is enriched with information through image processing and computer technology, and provides a more intuitive understanding with the human eye system. As a typical heterogeneous sensor image fusion, infrared and visible image fusion (IVIF) has already been applied in various fields, like military reconnaissance, video surveillance, vehicle night navigation, target detection and identification [1] and much more. There are special properties such as difference, complementarity, and correlation between different modalities of information. Infrared images can be captured by actively receiving the thermal radiation from objects, highlighting heat regions that are undetectable in the visible images, and working around the clock. However, infrared images are often blurred due to low spatial resolution. On the contrary, visible images exhibit high spatial resolution and rich texture details. Nevertheless, they are easily affected by weather factors, like rain, fog, or poor lighting [2,3]. IVIF techniques can make the best of the desirable characteristics of both imaging mechanisms. The aim of IVIF is to take advantage of the useful complementary information of multi-sensor images [4] and eliminate the possible redundancy and contradictions between them. As a result, the raw data can be used much more efficiently.

In the past few years, research on IVIF approaches has drawn extensive attention. In [5,6], researchers detailed many existing IVIF methods and analyzed their problems. Here, we further subdivide IVIF methods into three categories in accordance with the differences in fusion theory and architecture. We classify multi-scale transform (MST) methods [7], sparse representation (SR) methods [8], saliency-based methods [9], subspace-based methods [10], hybrid methods [11] and others [12,13] into the traditional methods. Deep learning methods employ a neural network to complete one or all of the three key steps (i.e., feature extraction, fusion and image reconstruction) involved in image fusion. According to the fused image acquisition process, this paper divides them into two modes: end-to-end image fusion methods and combinatorial-based image fusion methods. The end-to-end methods based on deep learning include the network architecture of autoencoder (AE), convolutional neural network (CNN) and generative adversarial network (GAN). Our method falls under this category. Another type is the combination of traditional and deep learning approaches, termed combinatorial-based methods. Examples include the combination of pulse coupled neural network (PCNN) and multi-scale transformation [14], the combination of CNN and Laplace pyramid decomposition [15], the combination of CNN and saliency-based [16], the combination of CNN and SR [17], etc., which are commonly used in image fusion tasks. While the above IVIF approaches have obtained impressive fusion performance, they still suffer from some drawbacks, especially in the traditional and combinatorial-based methods. The main problems with both methods lie in the following three folds. Firstly, it is quite challenging to design efficient image transformation and representation methods. The traditional methods adopt the same transformation and representation for heterogeneous images with multiple sources, resulting in the loss of differential information. Generally, image fusion methods have been explored to a large extent with the development of image representation theory. Therefore, it is urgent to investigate new image representation approaches to boost image fusion performance. Moreover, image decomposition is usually time-consuming. Secondly, designing complex activity-level measurements, feature extraction, or fusion operations in a manual manner will increase computing costs and algorithm complexity, further limiting their practicability. Thirdly, although deep learning techniques have been introduced into combinatorial-based methods, they are only performed for feature extraction or result reconstruction. Consequently, the limitations of traditional image fusion methods still remain.

In view of the above disadvantages, one research focus is to design IVIF models in an end-to-end fashion. In particular, the end-to-end methods completely circumvent the shortcomings of the traditional and combinatorial-based methods. For instance, DenseFuse [18] and TSFNet [19] utilize pre-trained AE architecture to extract features from source images and then reconstruct the fused images, which can achieve relatively promising fusion performance. DeepFuse [20] and RXDNFuse [21] are representative methods based on CNN, which can guide models to produce fused images via specially designed metrics of unsupervised learning. The FusionGAN [22], D2WGAN [23] and GANMcC [24] methods proposed based on GAN all apply adversarial games to reduce the difference in probability distribution between the fused images and the source images, and thus promote the preservation of original information.

Generally speaking, feature extraction and fusion of the source images are two key steps in the design of IVIF algorithms. On the basis of all previous comments, the motivation for our paper consists of two folds. In the first place, the key to image fusion is to design a more comprehensive feature extraction strategy based on neural networks. This is also the fundamental goal of training models for most IVIF algorithms, that is, to train a network with strong feature extraction capabilities. However, all of the above models only focus on single scale features in the sources. For example, Refs. [18,21,23] all operate at the same level of convolution kernel to extract specific scale features. Hence, the fusion results do not preserve the information of original features on a full scale. Additionally, a prerequisite for producing a fused image with highlighted targets and abundant texture information is the selection of important and salient features to be blended. Nevertheless, the handcrafted feature fusion strategies such as concatenation in channel-dimension or pixel-wise addition adopted by most IVIF methods cannot efficiently integrate significant features into fusion results in a way that is more consistent with human visual perception. As a result, the significant information in the sources is completely lost, and the reconstructed image has less gray level and low contrast.

To solve the problems mentioned above, we propose a novel IVIF method using GAN with multi-scale feature extraction (MFE) and joint attention fusion (JAF), called MJ-GAN. On one hand, multi-scale information in multimodality images is considered. More specifically, the highlighted objects in infrared images and the textures in visible images are automatically captured via more MFE modules. Additionally, an improved self-attention structure, which can achieve contextual information mining and attention learning, is introduced into MFEs to enhance the pertinence among multi-grained features. On the other hand, there is compelling evidence that the human visual system (HVS) automatically pays more attention to some salient features or areas rather than the whole. Therefore, we design a JAF network based on the channel attention and spatial attention to strengthen the attention to salient and important features in the source images during the feature fusion stage. Consequently, the fused images will be more consistent with human visual perception. Besides, it is also a well-known phenomenon that the stronger the discriminative ability of discriminators, the better the fused images produced by the generator. For this purpose, the loss functions of the dual discriminator are designed based on the idea of SCD loss function [25] to improve the discriminative ability of the discriminators. Specifically, we build the dual adversarial mechanism between the source images and their contributions to lessen the variance of the probability distribution between them.

**Figure 1:**
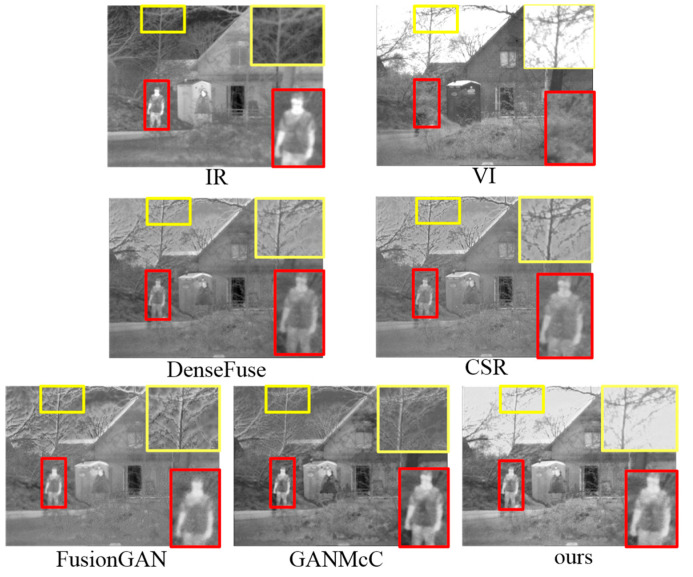
Illustration of the superiorities of our approach. The first row shows the infrared and visible images, the second and third rows are the fused images of DenseFuse, CSR, FusionGAN, GANMcC and our method, respectively.

To visually exhibit the superiorities of our method, we select some representative end-to-end methods, including DenseFuse [18], CSR [17], FusionGAN [22] and GANMcC [24] for comparison, as presented in Figure 1. Clearly, all comparison methods generate the fusion results with blurry thermal targets and insufficient textures, together with halos along the edges. By contrast, the fused image generated by our method keeps the high-contrast heat sources, reserves the richest and most natural background texture details, and accommodates human visual perception.

The contributions and characteristics of this work can be generalized as follows.

- To adequately preserve the global information, multi-scale feature extraction (MFE) modules are introduced into the two-stream structure-based generator to extract source image features of different scales for fusion.
- To focus more on the important and salient features during the fusion step, we select and merge significant features via a joint attention fusion (JAF) network.
- To improve the discriminative ability of the discriminator, a dual adversarial mechanism between the source images and their contributions is designed, which will drive the generator to transfer more original information into the final fused images.

The rest of our paper is organized as follows. We introduce some works in Section 2 that are closely associated with our method, including some end-to-end image fusion methods, an attention mechanism (AM) and FusionGAN. In Section 3, we present our algorithm’s details, including the overall framework of the proposed method, network architectures and loss functions. Plentiful comparison experiments on publicly available datasets are illustrated in Section 4. Additionally, we also implement generalization and ablation experiments in this section. Some concluding comments and an insightful discussion of our work are provided in Section 5.

## 2. Related Works

### 2.1. End-to-End Methods Used in Image Fusion

Currently, image fusion methods designed in an end-to-end manner have achieved fusion performance far exceeding traditional and combinatorial based methods. These methods mainly include CNN-based, AE-based, and GAN-based architectures. Especially for CNN- and GAN-based methods, the key steps in image fusion, namely feature extraction, feature fusion, and feature reconstruction, are implemented in an implicit fashion.

For the CNN-based approaches, infrared and visible image fusion (IVIF) are fulfilled by specially designed metrics of unsupervised learning. For instance, Long et al. [21] designed a new end-to-end method that combined the structural characteristics of ResNeXt and DenseNet to extract hierarchical features. Additionally, the loss function, defined as a combination of pixel-wise and feature-wise components, was minimized through the optimization of the pretrained VGG-19 network. STDFusionNet [26] was designed as an end-to-end method by introducing a salient target mask during training. In order to better guide extraction and reconstruction of the features, they elaborated a loss function that also incorporates the prominent target mask. Experimental results revealed that STDFusionNet could accomplish both highlighted object detection and critical information fusion. Prabhakar [20] firstly put forward a CNN-based unsupervised multi-exposure fusion algorithm, namely DeepFuse. But they could not extract much useful information from the source images due to the excessively simple network structure. All of the above CNN-based works highly rely on the ground truth of supervised learning or specially designed metrics of unsupervised learning. Therefore, they suffer from the following issues. Firstly, unlike image fusion in photography and remote sensing applications, the ground truth of infrared and visible fused images is essentially unavailable. Secondly, it is a challenge to design an efficient loss function to control fusion results. Thirdly, the structure of the designed network is too plain to extract more conspicuous features. Last but not least, thermal images are usually ignored by most CNN-based methods during the training stage, but are fed directly into the network trained on visible images during testing. So, the differences and associations between the sources have not yet been considered. To this end, the proposed model does not utilize CNN as the backbone architecture.

GAN has been a significant success in IVIF by virtue of the characteristics of its unsupervised adversarial learning manner, and over the last couple years with a number of effective algorithms being proposed [27]. In 2017, Ma et al. [22] reported the pioneering use of GAN for IVIF, namely FusionGAN. In the literature [28], a simple and effective relativistic discriminator was adopted to make the model converge quickly. More importantly, they innovatively utilized pre-fused images as the labels, which solved the problem of requiring ground truth in IVIF tasks. MgAN-Fuse [29] introduced multi-grained attention modules into the encoder–decoder to extract salient features, which addressed the problem of hardly perceiving the discriminative parts of an image existing in the previous GAN-based fusion methods. As an improved version, the multi-grained attention mechanism was further integrated into a generator and two discriminators in the Attention FGAN [30] model. Thus, the generator can focalize the most discriminative regions of the sources, and the discriminators can be constrained to focus more on the salient regions than on the entire input. Although the above GAN-based methods have achieved relatively good fusion results, there are still some shortcomings. Firstly, full-scale feature extraction is left out of consideration, resulting in the loss of global information in source images. Secondly, salient and important features cannot be selected for fusion, and a rough fusion operation is applied to fuse extracted features. Thus, the fused images are obtained in a manner inconsistent with human visual perception. Thirdly, the discriminator used in the above methods has a weak discriminative ability, which leads to reduced adversarial learning ability. Hence, the preservation of source information is inadequate.

Different from the methods based on CNN and GAN, the feature extraction and fused image rebuild in the AE-based methods are accomplished by pre-training an autoencoder model. But the feature fusion is implemented by applying some rough fusion strategies such as addition and L1-norm. For example, Li et al. [18] incorporated DenseNet [31] into the encoding network, which can extract more useful middle layer features that have been abandoned in other CNN-based models. In TSFNet [19], two independent encoders were used to extract discriminative features of diverse modalities. Yu Fu [32] came up with a dual-branch encoder structure to extract the semantic and detail information from the two source images, respectively. Han Xu [33] performed a coherent importance assessment of features of two source images by designing a pixel-wise classification saliency-based image fusion method (CSF) for the first time in a deep learning fashion. All of the above AE-based methods have demonstrated their powerful feature extraction capabilities; the proposed feature extractor also belongs to the AE architecture. Nevertheless, the handcrafted fusion rules used in the above methods are too coarse to preserve salient features.

Considering the above limitations, we accomplished IVIF using a novel GAN with multi-scale feature extraction (MFE) and joint attention fusion (JAF), together with two specifically stronger discriminators, which can achieve phenomenal fusion performance. Unlike previous approaches, the novelty of the proposed method lies in three points. First, we designed a multi-scale feature extraction module that is dedicated to extracting more comprehensive representations from the source images. Second, we designed a joint attention fusion module based on the spatial and channel attention mechanisms to recalibrate the extracted features and use them for fused image reconstruction. Third, according to the adversarial principle, a discriminator with better judgment can further force the generator to produce more realistic fusion results. Therefore, we designed two discriminators based on the principle of SCD (i.e., differential correlation sum), aiming at enhancing the discriminative ability of the proposed discriminators.

### 2.2. Attention Mechanism Used in Image Fusion

The attention mechanism (AM) has been widely used in various speech recognition [34], natural language processing [35] and computer vision [36] applications, due to its characteristic in accord with the human visual perception system. The principle of the AM is to calculate the weight between different regions or pixels according to their importance, so as to focus on the significant parts selectively. The basic idea of both multi-scale transform (MST) [7] and saliency-based [9] methods is to simulate the characteristics of the human visual system (HVS), that is, to focus more on the key information and ignore the unimportant information. In real-world scenarios, each target often contains components of different scales; the AM can selectively focus on typical regions within the image. Therefore, the performance of multimodality image fusion will be further improved when introducing the AM into the image feature extraction or fusion model. By incorporating the AM, the resulting fused image can reveal both the highlighted foreground object in infrared images and the abundant background textures in visible images.

### 2.3. GAN-Based Image Fusion Method

FusionGAN [22] was firstly proposed using GAN to fuse infrared and visible images, and our model is also designed based on it. In FusionGAN, infrared and visible images are firstly concatenated and then input into the generator (G) to generate fused images with major infrared heat-radiating information and few visible textures. In order to preserve the additional texture details in the visible image, a discriminator (D) is introduced. The adversarial interaction between a generator and a discriminator contributes to achieving this goal effectively.

The loss function of G in FusionGAN is formulated as:

(1) $$L_{G}=\frac{1}{N}\sum_{n=1}^{N}{\left(D(I_{f}^{n})-c\right)}^{2}+\frac{\lambda }{HW}({\left\|I_{f}-I_{r}\right\|}_{F}^{2}+\xi {\left\|\nabla I_{f}-\nabla I_{v}\right\|}_{F}^{2})$$

where N is the number of fused images, $I_{f}^{n}$ is the n-th fused image, $I_{r}$ and $I_{v}$ represent infrared and visible images, respectively, c denotes the soft label, H and W stand for height and width of the inputs, ${\left\|•\right\|}_{F}$ stands for matrix Frobenius norm, ∇ stands for gradient operator. λ is the hyperparameter to balance the adversarial loss of a G and a D (i.e., the first term on the right-hand side) and the content loss (i.e., the second term on the right-hand side). ξ is used to equilibrate the intensity similarity of fused images and infrared images and the gradient similarity of fused images and visible images.

The loss function of D in FusionGAN is formulated as follows:

(2) $$L_{D}=\frac{1}{N}\sum_{n=1}^{N}{\left(D(I_{v})-a\right)}^{2}+\frac{1}{N}\sum_{n=1}^{N}{\left(D(I_{f})-b\right)}^{2}$$

where a and b represent the soft labels, and ${D(I}_{v})$ and ${D(I}_{f})$ denote the judged results of visible images and fused images, respectively.

## 3. Proposed Method

### 3.1. Framework Overview

**Figure 2:**
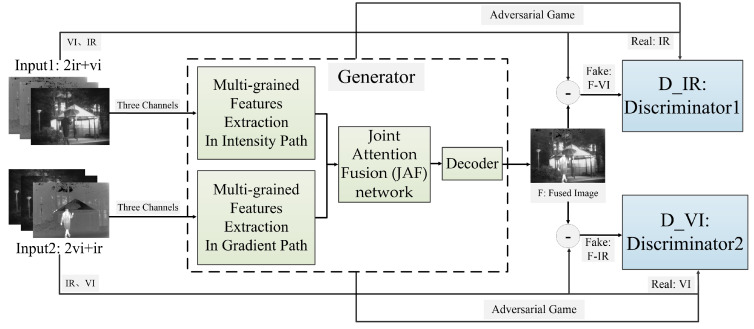
Our proposed method’s framework for IVIF.

In the training phase, the aim is to train a generator that can generate the preliminary fused image to fool the dual discriminator. The framework of our proposed method is schematically shown in Figure 2. Firstly, the two inputs of the proposed model are constructed by means of combining infrared and visible images in the difference ratio concatenation manner [37]. The inputs are then passed through two independent paths (i.e., intensity path and gradient path) to extract deep features at different scales via a multi-scale feature extraction (MFE) network. Secondly, the features extracted from the dual paths are recalibrated via the joint attention fusion (JAF) network, so that the final fusion results will preserve more important features. Thirdly, we can obtain a preliminary reconstructed fused image via a single decoder under the guidance of the hybrid loss function. Finally, the adversarial game with two enhanced discriminators (i.e., D_IR and D_VI) will compel the generator to preserve more meaningful information of both source images. Concretely, we input both sources and their contributions into the dual discriminator separately to discriminate which of the inputs are from source images. The above training process is repeated until neither discriminator can distinguish the contributions from the visible or infrared images. As a result, we obtain a well-trained generator capable of producing fused images containing more meaningful information. In other words, the combination of the generator with both MFE and JAF and enhanced discriminators can retain the salient features of the sources well, including prominent targets in infrared images and rich background textures in visible images.

In the testing phase, only the generator is working. Each image pair with arbitrary size as a whole from the test datasets is fed directly into the well-trained generator to produce the fused images with prominent thermal targets and perceptually pleasing backgrounds.

### 3.2. Model Architecture

#### 3.2.1. Generator Architecture

**Figure 3:**
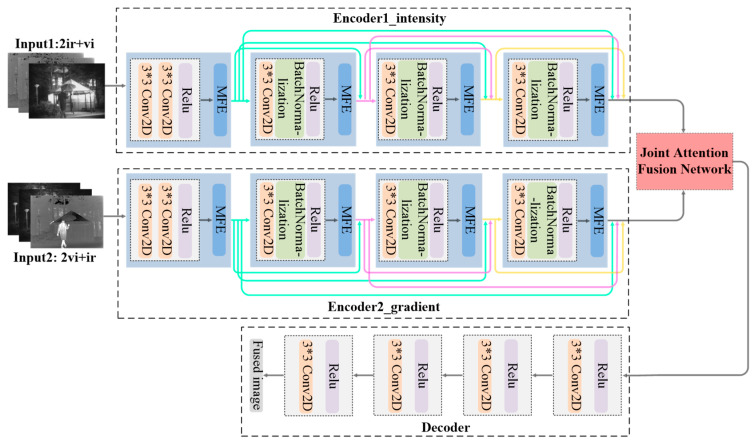
Schematic diagram of the generator.

Based on the requirement that achieving the leading fusion performance in the IVIF task should not only extract multi-scale features from the source inputs, but also choose important and salient features for fusion, the generator incorporates multi-scale feature extraction (MFE) modules and a joint attention fusion (JAF) network to achieve salient feature extraction and fusion. Also, the dual-encoder-single-decoder structure is adopted as the generator to achieve the cross-modality united representation and extraction of different information. Figure 3 shows its structure.

In the process of feature extraction, the MFEs are employed to extract multi-grained features from the source images. As we all know, the infrared image contains main contrast information and auxiliary texture information, while the primary texture information and secondary contrast information come from the visible image. The inputs obtained after concatenation are passed through two encoders separately to extract discriminative features at different scales from the source images. Then, in each path, an independent convolution block is first applied to roughly extract the shallow features from the input. Next, we can obtain the multi-scale features from the adjacent MFE block. Here, the combination of an independent convolution block and its neighboring MFE block is defined as a multi-scale block. In each encoding branch, four multi-scale blocks are arranged in turn to extract the deep features. To circumvent the problem of gradient vanishing caused by the deeper network designed in our model, the DenseNet structure is applied to each encoder. Benefiting from the dense connection, it can not only enhance the information flow between different multi-scale blocks, but also take full advantage of the multi-scale features extracted from the middle blocks. Finally, we can obtain hierarchical synthetic features for each modality image.

In the process of feature fusion, a JAF network based on spatial attention and channel attention is devised to select important features extracted by encoders for merging. Four simple but effective convolution blocks are employed in the reconstruction of the preliminary fused image. The structures of MFE and JAF will be described in detail in Section 3.2.1.1 and Section 3.2.1.2, respectively.

In the generator, two encoders share the same structure, which includes four 3×3 independent convolution blocks. Batch normalization is applied to all blocks except the first one to speed up convergence. The activation function is ReLU. The stride is fixed at 1 in all convolutional operations, and padding is used. Consequently, the inputs and outputs of the generator have the same size. Notably, all substructures used in the generator (i.e., dense connection, MFEs and JAF) can blend extracted features to some extent, so the utilization of complementary information will be greatly improved.

##### 3.2.1.1. Multi-Scale Feature Extraction (MFE) Architecture

**Figure 4:**
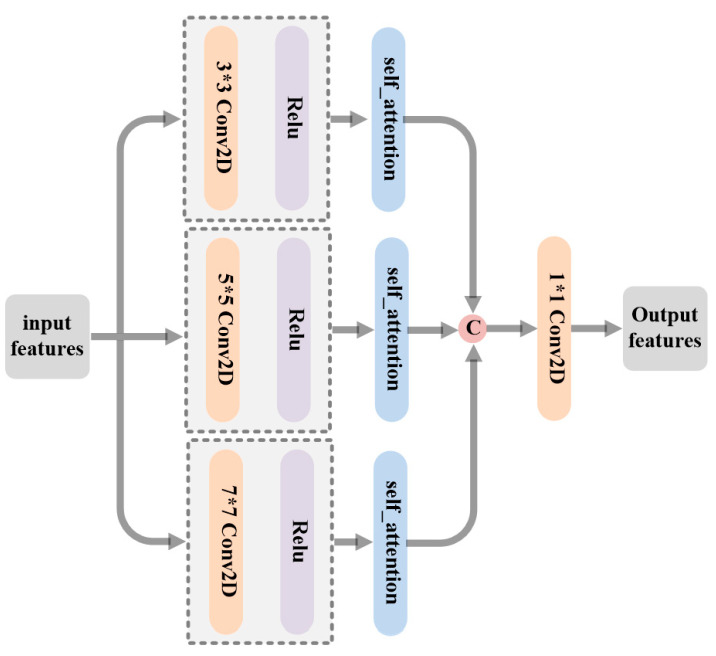
Schematic diagram of the MFE.

Efficient feature fusion depends on extracting comprehensive discriminative features from images of different modalities. As the single scale features extracted from the source inputs cannot represent the overall spatial properties of large targets in the original images, multiple convolution kernels with different sizes are used to extract comprehensive information from the sources. Figure 4 shows the structure of the MFE network.

The input features are fed into three separate branches containing 3×3, 5×5 and 7×7 filters followed by the ReLU activation layer to respectively extract image features from multiple scales. The resulting multi-scale features are expressed as follows:

(3) Fs1=Relu(Conv3×3(Finput))

(4) Fs2=Relu(Conv5×5(Finput))

(5) Fs3=Relu(Conv7×7(Finput))

where $F_{\mathrm{input}}$ represents the input of the MFE block, ${\mathrm{Conv}}_{*}$ represents the convolution operations with different scales, and $F_{s1}$, $F_{s2}$, $F_{s3}$ represent features extracted with different kernels sizes, respectively.

The application of multiple small convolution kernels can bring many benefits. On one hand, under the circumstance of an identical receptive field, the deeper the network, the more nonlinearity of the model. On the other hand, stacking multiple small filters can increase the receptive field, which also means that more global and intrinsic features of the target can be extracted. Not only that, but the parameters of the network will be greatly reduced. Therefore, instead of the 5×5 and 7×7 filters, two 3×3 kernels and three 3×3 kernels are the best choices, respectively.

**Figure 5:**
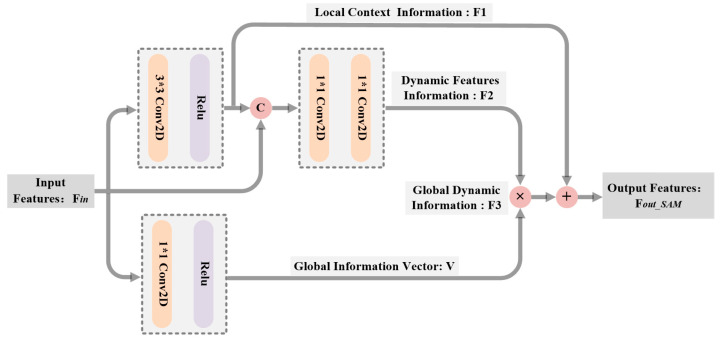
Schematic diagram of the improved self-attention block.

To address the redundancy and noise in multi-scale representations of source images, self-attention modules (SAM) are introduced in each branch, aiming to enhance important features. The major advantage of the SAM over other attention networks is that it can trigger interactions between different spatial locations to capture the intrinsic correlations of the input data. Hence, driven by [38], we propose an improved self-attention block that can achieve both contextual information mining and self-attention (SA) learning, so that it preserves salient features in both sources while suppressing insignificant features. Figure 5 shows the structure of the self-attention blocks contained in each MFE branch. In the global information capture path, the inputs, written as $F_{\mathrm{in}}$ for simplicity, are fed into an independent convolution block with 1×1 filters to output a vector (written as V). Thus, the extracted global static information can be formulated as follows:

(6) V=Relu(Conv1×1(Fin))

The purpose of the other path is to extract dynamic feature information. The original inputs $F_{\mathrm{in}}$ are firstly fed into a convolution block with 3×3 kernels to acquire local context information (written as $F_{1}$), which can be expressed as follows:

(7) F1=Relu(Conv3×3(Fin))

To deepen the interaction between features, we also concatenate $F_{1}$ with original inputs in the channel dimension. And then, two sequential convolution blocks with 1×1 filters are used to learn dynamic feature information (written as $F_{2}$), which is expressed as follows:

(8) $$F_{2}=Conv_{1\times 1}(Conv_{1\times 1}(\left\lfloor F_{in},F_{1}\right\rfloor ))$$

Hence, the feature represented by global dynamic information (written as $F_{3}$) can be obtained:

(9) $$F_{3}=V\times F_{2}$$

The enhanced feature via SAM is then gained by fusing local static feature $F_{1}$ and global dynamic feature $F_{3}$:

(10) $$F_{out\_SAM}=F_{1}+F_{3}$$

Finally, for integrating the multi-grained feature information, the outputs of each branch are first concatenated and then passed through a convolution layer with a 1×1 kernel. Consequently, the aggregated multiscale features are expressed as follows:

(11) $$F_{out}=Conv_{1\times 1}(\left\lfloor self(F_{s1}),self(F_{s2}),self(F_{s3})\right\rfloor )$$

where $F_{\mathrm{out}}$ stands for the output of the MFE block, self(•) represents the self-attention enhancing modules, and $\left\lfloor •\right\rfloor$ denotes the concatenation operation.

##### 3.2.1.2. Joint Attention Fusion (JAF) Architecture

**Figure 6:**
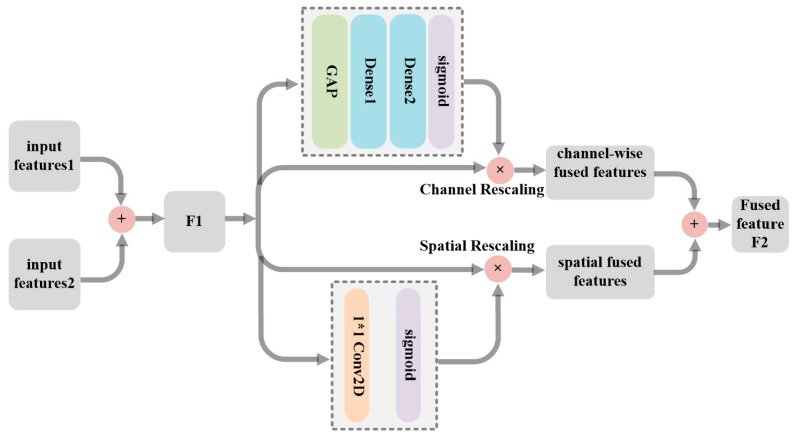
Schematic diagram of the JAF.

Crucially, some salient and important features extracted from the dual encoder are automatically chosen and then integrated into a single new image for achieving state-of-the-art performance in IVIF. By coincidence, this idea can be implemented by an attention mechanism (AM). The AM works by accessing all input sequences to calculate weights, then combining the weights with the inputs to selectively strengthen the attention more toward discriminant parts of the input images. Therefore, motivated by the success of AM in IVIF [39], a joint attention fusion (JAF) network is constructed in parallel by channel attention and spatial attention to merge the extracted features. Figure 6 shows the schematic diagram of the JAF. As the dual encoders have the same structure, the features extracted from them are firstly added to obtain the initial fused features, written as F1. Subsequently, the performance of feature merging is further improved via channel-attention and spatial-attention networks, respectively.

In the channel dimension, we mainly focus on which features of the input are meaningful. In general, the infrared radiation information is mainly represented by the low-frequency information captured by a global average pooling operation. Therefore, the inputs F1 with h×w×c are firstly transformed into a 1×1×c compressed channel representation using the global average pooling operation to obtain the global information of the given input features. Next, the importance of each channel is learned via two fully connected (FC) layers, and then the weight coefficients are calculated through the sigmoid activation function. Finally, the recalibration features (i.e., the channel-wise fused features) can be obtained by multiplying the weight coefficients by the initial fused feature F1. Consequently, the channel-attention network can selectively emphasize important features while suppressing others. The process of using channel attention to enhance features can be shown in Equation (12):

(12) $$F_{out\_chan}=(Sig(FC_{2}(FC_{1}(GAP(F_{1})))))\times F_{1}$$

where $F_{\mathrm{out}\_\mathrm{chan}}$ represents the recalibration feature of the channel attention, GAP represents the global average pooling operation, and Sig denotes the sigmoid function.

In the spatial dimension, the purpose is to concentrate on which parts of the output are rich in effective information, which can bridge the shortage of only using channel attention to some extent. We utilize a simple yet effective 1×1 convolution layer to learn the importance of each input feature F1. Similarly, the weight coefficients are also obtained by sigmoid, which means the amount of information contained in each feature. At last, the informative features (i.e., the spatial fused features) can be obtained by multiplying the weight coefficients by the initial fused feature F1. The process of using spatial attention to enhance features can be shown in Equation (13):

(13) $$F_{out\_spat}=(Sig(Conv_{1\times 1}(F_{1})))\times F_{1}$$

where $F_{\mathrm{out}\_\mathrm{spat}}$ represents the recalibration feature of the spatial attention.

To sum up, the purpose of selecting salient features to reconstruct a fused image can be achieved by re-mixing channel-wise fused features and spatial fused features:

(14) $$F_{fuse}=F_{out\_chan}+F_{out\_spat}$$

#### 3.2.2. Dual-Discriminator Architecture

**Figure 7:**
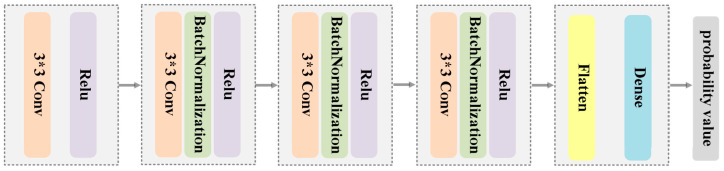
Schematic diagram of the discriminator.

In our work, the two enhanced discriminators have the same structure. Figure 7 presents the architecture of the discriminator, which consists of four convolutional blocks. A convolution layer with 3×3 kernel and ReLU is applied in the first four blocks. It is worth noting that all convolution blocks except the first one employ batch normalization operations to improve the convergence speed. Subsequently, the flattened data are fed into a full-connection layer that outputs a scalar value of the estimation probability. Unlike the generator, the stride of each convolution layer is 2 and has no padding.

### 3.3. Loss Function

#### 3.3.1. Loss Function of Generator

To guide model training in an even better fashion, the loss function of the generator considers structural information, complementary information (i.e., primary and secondary information contained in sources) of multi-mode images, and the adversarial losses between a generator and two discriminators. We formalize it as follows:

(15) $$L_{G}=\lambda_{1}L_{content}+\lambda_{2}L_{SSIM}+\lambda_{3}L_{adv}$$

where $\lambda_{1}$, $\lambda_{2}$ and $\lambda_{3}$ are the positive parameters to control the trade-off among three items, respectively.

$L_{\mathrm{content}}$ represents the content loss. In order to address the under-utilization of information and inspired by [24], $L_{\mathrm{content}}$ is designed as follows:

(16) $$\begin{array}{l} L_{content}=\varepsilon_{1}{\left\|I_{fused}-I_{ir}\right\|}_{2}+\varepsilon_{2}{\left\|I_{fused}-I_{vi}\right\|}_{2}+\\ \varepsilon_{3}{\left\|\nabla I_{fused}-\nabla I_{ir}\right\|}_{2}+\varepsilon_{4}{\left\|\nabla I_{fused}-\nabla I_{vi}\right\|}_{2} \end{array}$$

where $I_{\mathrm{fused}}$, $I_{\mathrm{ir}}$ and $I_{\mathrm{vi}}$ are the fused images, the infrared images, and the visible images, respectively. ${\left\|•\right\|}_{2}$ denotes 2-norm, and ∇ represents gradient operator. $\varepsilon_{1}$, $\varepsilon_{2}$, $\varepsilon_{3}$ and $\varepsilon_{4}$ are the weights used to balance the above items.

To measure the loss of structural integrity as well as luminance consistency, the structural similarity loss $L_{\mathrm{SSIM}}$ is introduced. For IVIF, the formula definition is:

(17) $$L_{\mathrm{SSIM}}=(1-\mathrm{SSI}M_{I_{fused},I_{ir}})+\eta (1-\mathrm{SSI}M_{I_{fused},I_{vi}})$$

where ${\mathrm{SSIM}}_{\left(•\right)}$ stands for the structural similarity measure between two images (i.e., the fused image and two source images). η is used to achieve an equilibrium between them.

The adversarial loss $L_{\mathrm{adv}}$ is defined as follows:

(18) $$L_{adv}=\frac{1}{N}\sum_{n=1}^{N}{\left(D_{ir}(I_{fused})-a\right)}^{2}+\frac{1}{N}\sum_{n=1}^{N}{\left(D_{vi}(I_{fused})-a\right)}^{2}$$

where D(•) stands for the estimated result of the two discriminators. Due to the expectation of the generator that the discriminators will judge the fused image as real data, the soft label of a ranges from [0.7, 1.2].

#### 3.3.2. Loss Function of Discriminator

Indeed, according to the loss function of the generator designed in this article, the fusion results with both the information of heat radiation and visible textures can be obtained in the absence of a discriminator. But that is far from enough. Therefore, the adversarial architecture is adopted to keep more information of the sources in the fusion results. Generally speaking, the stronger the discriminative ability of the discriminator, the better the implementation of the fused image produced by the generator. We thus designed two discriminators with the same simple and naïve structure to respectively distinguish one of the source images and its contribution [40]. Specifically, we input $\left|F-S_{2}\right|$ and $S_{1}$ to the first discriminator (i.e., D_IR) and feed $\left|F-S_{1}\right|$ and $S_{2}$ into the second discriminator (i.e., D_VI) to make it difficult for the discriminators to distinguish the inputs. Hence, the adversarial relationship between the two discriminators and a generator is stronger.

The loss function of the dual discriminator can be denoted as:

(19) $$L_{D_{1}}=\frac{1}{N}\sum_{i=1}^{N}{\left(D(\left|I_{fused}-I_{ir}\right|)-b\right)}^{2}+\frac{1}{N}\sum_{i=1}^{N}{\left(D(I_{vi})-a\right)}^{2}$$

(20) $$L_{D_{2}}=\frac{1}{N}\sum_{i=1}^{N}{\left(D(\left|I_{fused}-I_{vi}\right|)-b\right)}^{2}+\frac{1}{N}\sum_{i=1}^{N}{\left(D(I_{ir})-a\right)}^{2}$$

where N denotes the number of images (i.e., the sources or the fused images). D(•) denote the classification results. $\left|I_{\mathrm{fused}}-I_{\mathrm{ir}}\right|$ represents the contribution of the source visible images, and $\left|I_{\mathrm{fused}}-I_{\mathrm{vi}}\right|$ indicates the contribution of the source infrared images. Both of the above represent false data and will be reduced by the discriminators. Nevertheless, the source image $I_{\mathrm{ir}}$ and $I_{\mathrm{vi}}$ will be increased. Thus, the soft label of b is in the range of [0, 0.3].

## 4. Qualitative and Quantitative Experiments

### 4.1. Experiment Details

In this section, we will firstly introduce four publicly available datasets in detail for training and testing the proposed model. Then, the details of training and testing are described. Thirdly, we choose nine state-of-the-art algorithms for comparison with our method. Finally, we introduce eight commonly used metrics for quantitative evaluation of image fusion performance.

#### 4.1.1. Publicly Available Datasets

We selected three commonly used IVIF datasets to train or test our method, including the TNO dataset (available at https://figshare.com/articles/dataset/TNO_Image_Fusion_Dataset/1008029) (accessed on 5 February 2023), OSU dataset (available at http://vcipl-okstate.org/pbvs/bench) (accessed on 5 February 2023) and RoadScene dataset (available at https://github.com/hanna-xu/RoadScene) (accessed on 5 February 2023). The TNO dataset mainly takes military scenes as background and collects infrared and visible image pairs of soldiers, vehicles, and buildings in different environments under the same scene for image fusion research. The OSU dataset includes infrared grayscale images and color visible image sequences. As we only studied grayscale image fusion, we needed to convert color images to grayscale images in advance. The RoadScene dataset is dominated by traffic scenes, including different scenes formed by the combination of roads, cars, pedestrians, buildings, and other elements. Compared with the TNO dataset, it has higher spatial resolution. The above three datasets all contain pairs of normally exposed infrared and visible images. It is well known that the goal of infrared and visible image fusion is to generate fused images with significant targets and rich textures in extreme environments. Therefore, we further tested the robustness of the proposed algorithm on the Multi-Spectral Road Scenarios (MSRS) dataset (available at https://github.com/Linfeng-Tang/MSRS) (accessed on 5 February 2023) containing both night and daytime scene images. The MSRS dataset contains 1444 pairs of aligned infrared and visible images of high quality. Each dataset and its role are clarified below.

During training, 55 infrared and visible image pairs were selected from the TNO dataset, which included the images with different scenes and resolutions that had been registered [29]. However, these training data were insufficient to train our IVIF model. We adopted the common expansion strategy of non-overlapping clipping to expand the training image samples. Concretely, a stride was set as 12 and then an 84 × 84 patch was randomly cropped from each image pair. Eventually, 61,679 image patches were obtained to train the proposed model. All training data were scaled to the range of −1 to 1.

During testing, we also selected an additional 38 image pairs from the TNO dataset to carry out the fusion performance verification of our algorithm. Additionally, the generalization capabilities of the deep-learning model are also an important way to evaluate the robustness of algorithms. Therefore, the commonly used image fusion datasets, i.e., OSU, RoadScene and MSRS datasets were picked, and we chose 24, 20 and 40 infrared and visible image pairs from them, respectively. It is worth noting that as we only studied IVIF in single-band gray, additional preprocessing should be performed for the aforementioned datasets to satisfy the experimental requirements.

#### 4.1.2. Training Details and Parameter Settings

Training details: During the training stage, to maintain adversarial relationships between the two types of networks, we initially trained the dual discriminator (i.e., D_IR and D_VI) three times (t = 3) before alternating the training of the generator (G) and dual discriminator once per batch. As the training process continued, more infrared intensity information and visible texture information were gradually added to the fusion result when the dual discriminator could hardly distinguish the source images from its contributions simultaneously. In other words, we obtained a generator with strong capability that could produce realistic fused images. The entire training details are presented in Algorithm 1. During the testing stage, only the G was valid. Each image pair from the training dataset or testing datasets was fed into G as a whole instead of image patches to directly generate the fusion result. The proposed network was programmed on TensorFlow.
**Algorithm 1:** Our model’s training details
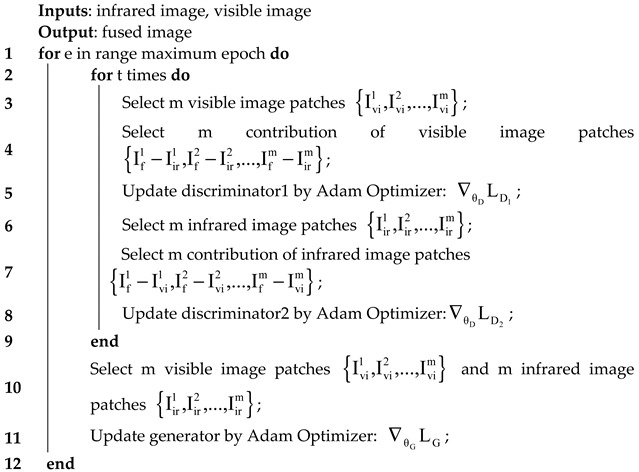



Parameter settings: The learning rates of G and D were the same as $1\times 10^{-5}$, batch size was fixed at 20, epoch was set at 10, and the optimizer was Adam. The other parameters in the loss function of G were set as $\lambda_{1}{=\lambda }_{3}=1$, $\lambda_{2}=0.35$, $\varepsilon_{1}{=1,\varepsilon }_{2}=0.3$, $\varepsilon_{3}{=3,\varepsilon }_{4}=5$, η=1.8.

#### 4.1.3. Baseline Methods

As described in Section 3, several subnetwork structures are involved in our model based on the GAN architecture, such as encoder–decoder and DenseNet. Therefore, the aforementioned subnetworks included in IVIF algorithms should be competitors to be compared with our method. Methods containing DenseFuse [18], FusionGAN [22], GANMcC [24], Dualbranch [32] and CSF [33] were recently introduced with the aim of achieving state-of-the-art fusion results. Additionally, to capture original image information from multiple scales to improve the perception of HVS, methods that most commonly make use of sparse representation (SR) and multi-scale transform (MST) have also been introduced. Hence, some traditional and combinatorial-based representative competitors, including convolutional sparse representation (CSR) [17], discrete cosine harmonic wavelet (DCHWT) [41], multi-resolution singular value decomposition (MSVD) [42], and multi-scale weighted gradient-based fusion (MWGF) [43], were also compared against ours. In conclusion, to prove the superiority of our method, nine mainstream methods were selected to compare fusion performance qualitatively and quantitatively with ours on four public datasets. The idea behind these experiments was to combine the merits of all previous approaches while avoiding the shortcomings of each.

To guarantee the fairness of the evaluation results, all competing methods were run based on the publicly available code of the corresponding author or a well-known toolbox, and the settings refer to corresponding original papers. The above methods were run on the same GPU, an NVIDIA GeForce RTX 3070.

#### 4.1.4. Objective Metrics

Trivial differences among fusion results will bring about a challenge for precise subjective assessment. Generally, it is a reasonable matter to adopt multiple image quality metrics for overall evaluation. Over the past few years, all kinds of quantitative assessment indexes for IVIF algorithms have been presented [44]. These metrics can be categorized as information theory-based, structural similarity-based, image features-based, human visual perception-based, and sources and fused images-based. But to be honest, none of them is certainly better than the others. Hence, a multi-index evaluation system covering the above quality indicators was adopted in this work to perform a thorough evaluation of the generated fused images. Herein, we selected eight commonly used metrics to evaluate our model quantitatively, including sum of the correlations of differences (SCD) [25], mutual information (MI) [45], correlation coefficient (CC) [46], standard deviation (SD) [47], spatial frequency (SF) [48], visual information fidelity for fusion (VIFF) [49], mean structural similarity index measurement (MSSIM) [49], and entropy (EN) [50]. Each of them is described in detail below.

(1) SCD

SCD can be adopted to measure how much of the fused image comprises complementary information from the two source images. The differences between the fused image (F) and two sources (S_1_, S_2_) can be formulated as:

(21) $$D_{1}=F-S_{2}$$

(22) $$D_{2}=F-S_{1}$$

The SCD can be expressed as:

(23) $$SCD=r(D_{1},S_{1})+r(D_{2},S_{2})$$

where r(•) is to calculate the similarity between $D_{k}$ and $S_{k}$, which is defined as:

(24) $$r(D_{k},S_{k})=\frac{\sum_{i}\sum_{j}\left(D_{k}(i,j)-\overset{-}{D_{k}})(S_{k}(i,j)-\overset{-}{S_{k}}\right)}{\sqrt{\sum_{i}\sum_{j}{\left(D_{k}(i,j)-\overset{-}{D_{k}}\right)}^{2}\sum_{i}\sum_{j}{\left(S_{k}(i,j)-\overset{-}{S_{k}}\right)}^{2}}}$$

where $\overset{-}{D_{k}}$ and $\overset{-}{S_{k}}$ stand for the average of the pixel values of $D_{k}$ and $S_{k}$.

(2) MI

MI estimates the amount of information transferred from the two source images to the fused image. The definition of MI in infrared and visible image fusion is:

(25) $$MI_{X,F}=\sum_{x,f}p_{X,F}(x,f)\log_{2}\frac{p_{X,F}(x,f)}{p_{X}(x)p_{F}(f)}$$

(26) $$MI=MI_{v,f}+MI_{r,f}$$

where $p_{X}\left(x\right)$ and $p_{F}\left(f\right)$ represent the edge histograms of the images X and F, respectively. $p_{X,F}\left(x,f\right)$ represents the joint histogram of the images X and F. ${\mathrm{MI}}_{r,f}$ denotes the MI value between the infrared image and the fused image, when the infrared image is taken as reference. Similarly, ${\mathrm{MI}}_{v,f}$ represents the MI value between the visible image and fused image, when the visible image is taken as reference. The sum of the two MI values equals the final MI value.

(3) CC

CC can measure the degree of linear correlation between the sources and the fused image. It is mathematically expressed as:

(27) $$CC=\lambda_{a}r_{v,f}+\lambda_{b}r_{i,f}=\lambda_{a}\frac{\sum_{i=1}^{M}\sum_{j=1}^{N}\left(V_{i,j}-\mu_{V})(F_{i,j}-\mu_{F}\right)}{\sqrt{\sum_{i=1}^{M}\sum_{j=1}^{N}{\left(V_{i,j}-\mu_{V}\right)}^{2}\sum_{i=1}^{M}\sum_{j=1}^{N}{\left(F_{i,j}-\mu_{F}\right)}^{2}}}+\;\lambda_{b}\frac{\sum_{i=1}^{M}\sum_{j=1}^{N}\left(I_{i,j}-\mu_{I})(F_{i,j}-\mu_{F}\right)}{\sqrt{\sum_{i=1}^{M}\sum_{j=1}^{N}{\left(I_{i,j}-\mu_{I}\right)}^{2}\sum_{i=1}^{M}\sum_{j=1}^{N}{\left(F_{i,j}-\mu_{F}\right)}^{2}}}$$

where $\mu_{V}$, $\mu_{I}$ and $\mu_{F}$ denote the mean values of the two sources and the fused image, respectively.

(4) SD

SD can express the contrast of the fused image. The definition of the SD is:

(28) $$SD=\sqrt{\frac{1}{MN}\sum_{i=1}^{M}\sum_{j=1}^{N}{\left(F_{i,j}-\mu \right)}^{2}}$$

where $F_{i,j}$ is the pixel value of the fused image with the size of M×N at the point (i,j), and μ is the average pixel value of the fused image.

(5) SF

SF can reflect the texture details of the fused image according to gradient distribution and is defined by spatial row frequency and column frequency:

(29) $$RF=\sqrt{\sum_{i=1}^{M}\sum_{j=2}^{N}{\left(F_{i,j}-F_{i,j-1}\right)}^{2}}$$

(30) $$CF=\sqrt{\sum_{i=2}^{M}\sum_{j=1}^{N}{\left(F_{i,j}-F_{i-1,j}\right)}^{2}}$$

(31) $$SF=\sqrt{RF^{2}+CF^{2}}$$

(6) VIFF

VIFF is used to calculate the fidelity of the fused image based on human visual perception and is formulated as:

(32) $$VIFF(X,F)=\frac{\sum_{k}\sum_{b}\log_{2}(1+\frac{g_{k,b}^{2}{\left(\sigma_{k,b}^{X}\right)}^{2}}{\left({\left(\sigma_{k,b}^{F}\right)}^{2}-g_{k,b}^{2}•{\left(\sigma_{k,b}^{X}\right)}^{2}+\sigma_{N}^{2}\right)})}{\sum_{k}\sum_{b}\log_{2}(1+\frac{{\left(\sigma_{k,b}^{X}\right)}^{2}}{\sigma_{N}^{2}})}$$

where $g_{k,b}=\frac{\sigma_{k,b}^{X,F}}{{{(\sigma }_{k,b}^{X})}^{2}}$. X and F are the source images and the fused image, respectively. $\sigma_{N}$ is the hypothetical covariance of the VIFF function. $\sigma_{k,b}^{X}$ represents the standard deviation of the b-th sub-band of the k-th image block of the sources. $\sigma_{k,b}^{X,F}$ denotes the covariance of the sources.

In practice, the calculation steps include: (1) filter and divide the source images and the fused image into different blocks; (2) evaluate the visual information of each block; (3) calculate the VIF for each sub-band; (4) calculate the overall index.

(7) MSSIM

SSIM is used to model loss and distortion between the sources and fused image based on their similarities in light, contrast, and structure information. Mathematically, MSSIM can be defined as follows:

(33) $$SSIM(X_{i},F_{i})=\sum_{X_{i},F_{i}}\frac{2\mu_{X_{i}}\mu_{F_{i}}+c_{1}}{\mu_{X_{i}}{}^{2}+\mu_{F_{i}}{}^{2}+c_{1}}•\frac{2\sigma_{X_{i}}\sigma_{F_{i}}+c_{2}}{\sigma_{X_{i}}{}^{2}+\sigma_{F_{i}}{}^{2}+c_{2}}•\frac{\sigma_{X_{i}F_{i}}+c_{3}}{\sigma_{X_{i}}\sigma_{F_{i}}+c_{3}}$$

(34) $$MSSIM(V,R,F)=\frac{1}{2M}(\sum_{i=1}^{M}SSIM(V_{i},F_{i})+\sum_{i=1}^{M}SSIM(R_{i},F_{i}))$$

where μ denotes the mean value of the corresponding images, σ is the standard deviation of the corresponding images, and $c_{1}$, $c_{2}$ and $c_{3}$ are the constant values to make the algorithm stable, respectively.

(8) EN

EN is used to measure the amount of information in the fused image. The mathematical formula of EN is expressed as:

(35) $$EN=-\sum_{l=0}^{L-1}p_{l}\log_{2}p_{l}$$

where L represents the gray level of the fused image, and $p_{l}$ is the normalized histogram with the gray level of l in the fused image.

What is noteworthy is that the higher the above metrics, the better the fused image. Moreover, we used the codes provided by the author or a well-known third party to perform the calculations of all image quality indicators through MATLAB.

### 4.2. Results in TNO Dataset

#### 4.2.1. Qualitative Analysis

**Figure 8:**
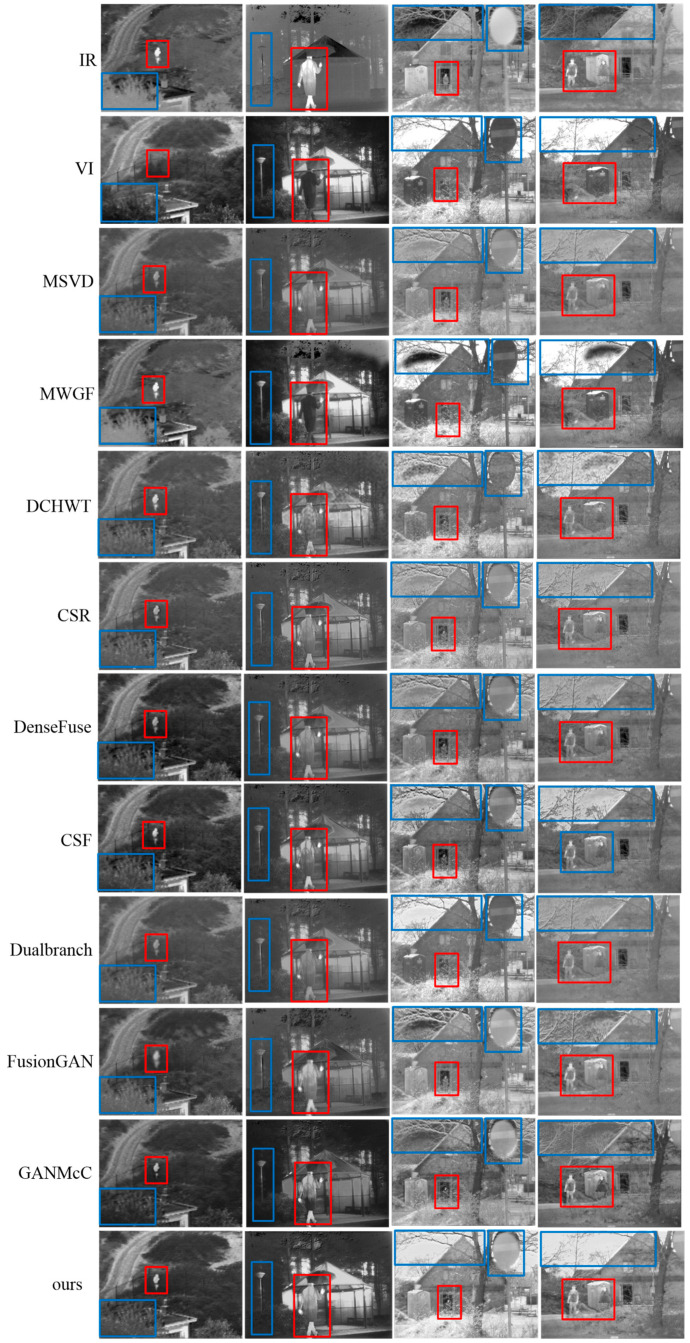
Intuitive analysis of our method and nine leading methods on the TNO dataset.

At first, four infrared and visible image pairs from the TNO dataset were selected to implement fusion operations using different methods. As shown in Figure 8, there were some visual results for the fusion performance. The infrared images described the scene that showed hot objects well, e.g., pedestrians, while the abundant background details were provided by the visible images, such as grass clusters, street lamps, and tree branches. The ideal fused image should contain both prominent thermal targets and rich background textures and be artifact-free. In other words, the fused image should resemble an infrared image as well as a visible image. We marked some distinctive areas with different color frames in the sources and the fused images for easier observation.

From Figure 8, we can see that almost all comparison methods can achieve certain fusion results. However, neither of the above two tasks (i.e., the resulting fused image should retain both salient targets and rich texture details without introducing any artifacts) was well achieved. The MSVD method generated the fusion results with low brightness, such as blurred pedestrian targets in the fusion results. This demonstrated that more features could be extracted from the two sources by the MSVD method, but the visible features diluted the thermal radiation information during the feature fusion process, resulting in less prominent heat sources. The MWGF method could well obtain more information from the sources, but undesirable visual artifacts were introduced (such as unnatural artifacts and noise in the background of the fusion results). This is because the introduction of more spectral information from infrared images into fused images will destroy the visual quality of visible images. To make matters worse, it seems that the hot target information in the fused images was barely drawn from the infrared images. The fusion results produced by the DCHWT method also had the problem of texture information destruction as well as unnatural visual experiences to some extent, such as pedestrians and background branches in the fusion results. The CSR and DenseFuse methods could extract the thermal targets from the source images well, such as persons. However, some regions, such as the pedestrians in the fused images, showed low brightness and unhighlighted thermal targets due to the image energy loss. The fusion results of the CSF could not highlight the thermal targets. Due to information loss caused by downsampling, the fusion results of the Dualbranch were blurred. The targets extracted by the FusionGAN method had a halo effect along the edges. The reason is that the FusionGAN method does not account for additional thermal information. Additionally, FusionGAN reconstructed the sky scene of the fused image with unnatural artifacts. The GANMcC method achieved results comparable with ours, but smoothed out most of the textures of the fused images, leading to low contrast of the fused images. After intuitive comparative analysis, we could see that our method acquired excellent performance in terms of thermal object extraction and comparable of background texture details.

#### 4.2.2. Quantitative Analysis

We selected 37 image pairs from the TNO dataset to objectively assess the fusion performance of our method. Table 1 lists the measurement results for different fusion methods using eight image quality metrics. Bold-red and bold-blue values indicate the best and second-best values for that column metric by the corresponding row algorithm compared to the others, respectively. Obviously, our method achieved the highest average values on six measurements, i.e., EN, MSSIM, SD, VIF, CC and SCD. Our method’s results for the remaining two metrics merely followed behind GANMcC and MWGF by a slight margin, respectively.

The first and second ranks on EN and MI denote that the fusion results of our method contained the maximum amount of information. The best and second-best SD and SF demonstrated that our fusion results for the highest contrast contained more edges and texture details. The best MSSIM showed that the fusion results of our method had the highest structural similarity to the two source images, and implied the least loss and anamorphosis in our fused images. The best VIFF indicated that the fusion results for the proposed method were more in line with the HVS. The best SCD and CC values indicated that our fused images were highly correlated with the source images. All in all, the performance of our method was competitive on all eight metrics. Among the quantitative evaluation metrics of the fusion results obtained by our method, few were lower than those of the other comparison algorithms. This is because our method achieved multi-scale representation of the source images and selected significant features to reconstruct the fused images.

### 4.3. Generalization Results in OSU Dataset

#### 4.3.1. Qualitative Analysis

**Figure 9:**
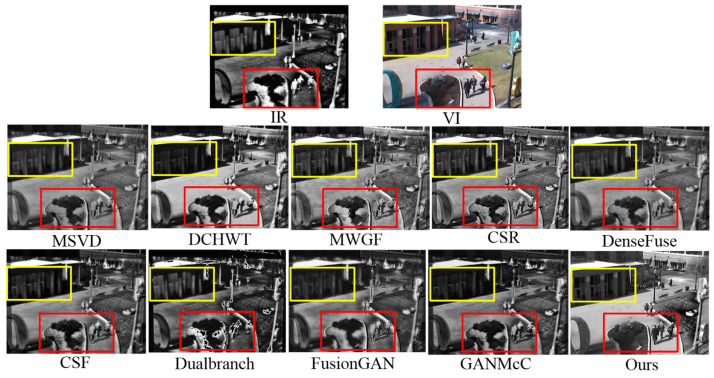
Intuitive analysis of our method and nine leading methods on the OSU dataset.

We validated the generalization ability of the proposed method on the OSU dataset, and Figure 9 shows the comparison results. In the OSU dataset, the infrared images contained radiating targets, such as pedestrians and parterre marked with red rectangles, while the visible images had rich details and high visual perception, such as buildings marked with yellow rectangles. Apart from ours, almost all fused images suffered from unpleasant artifacts that caused degradation of the visual quality. Clearly, our method performed better than others in terms of thermal target extraction, spatial detail retention and visual perceptual quality.

#### 4.3.2. Quantitative Analysis

Table 2 shows a quantitative comparison between the proposed method and its competitors after examination of the fusion results by subjective evaluation. The values among all methods shown as bold red, bold blue and bold green denote the best, second-best and third-best scores, respectively. Clearly, the fusion performance of our method was in the top place on the six metrics, i.e., MI, SF, SD, VIFF, CC, SCD. The scores on the EN metric were suboptimal. It can be inferred that our method’s fusion performance was phenomenal in terms of information retention, visual quality, and correlation with two source images. Although the results for the MSSIM were lower than those obtained with the CSR and DenseFuse, there is no doubt that our method achieved the best performance on all indexes. This shows that our method is robust on the OSU dataset.

### 4.4. Generalization Results in RoadScene Dataset

#### 4.4.1. Qualitative Analysis

**Figure 10:**
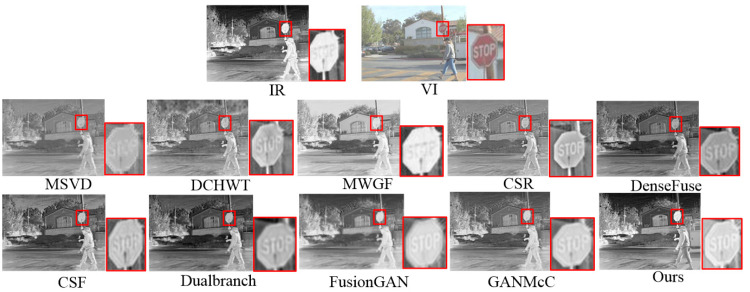
Intuitive analysis of our method and nine leading methods on the RoadScene dataset.

The RoadScene dataset is also commonly used in IVIF tasks. Therefore, 20 image pairs from the RoadScene dataset were selected to implement the test of generalization ability. Figure 10 shows one of the generalized results for the different methods. The infrared images contained heat source targets and spatial textures, while the visible images exhibited better visual perception. For easier observation, distinct regions in the source images and the fusion results were marked with red rectangles. They were subsequently enlarged and placed in the lower right corner. As we can see, both the MSVD and MWGF methods failed to extract more spatial textures, leading to artifacts such as marker regions in the fused images. Although the DCHWT method generated the fusion result with higher contrast, it also introduced artifacts in the sky. The fusion result generated by the CSR method had a high structural similarity with the source images, but some details were still lost. The DenseFuse method preserved details well, but the fused image suffered from low brightness. Although the results with the CSF contained rich information, the low contrast resulting in blurred signs. Due to the low brightness, the fused image generated by the Dualbranch method showed a black appearance. The FusionGAN method produced a fused image with halo effects along the target edges. The GANMcC method produced a fused image that contained more target information and spatial textures, but the contrast was relatively low. To a certain degree, we can say that our algorithm provided a more pleasing fused image with clearer texture details, better visual quality, and higher contrast.

#### 4.4.2. Quantitative Analysis

In order to further verify the generalization ability of our method, 20 image pairs from the RoadScene dataset were calculated by eight evaluation metrics to analyze quantitatively. Table 3 shows that the performance of our approach on the EN, SF, CC, and SCD metrics was best and fell behind the DCHWT and CSF methods by a narrow margin on the SD and VIFF metrics. Our fusion results had the highest CC and SCD values, and it could be verified that the fused images generated by our method were visually more like the sources. The best EN and SF values verified that our results retained more information. Although the results on the MI and MSSIM metrics were inferior to the second-best score, they also provided competitive fusion results. The competitive evaluation results indicate that the MFE and JAF modules in our model still worked well on the RoadScene dataset.

### 4.5. Generalization Results in MSRS Dataset

#### 4.5.1. Qualitative Analysis

**Figure 11:**
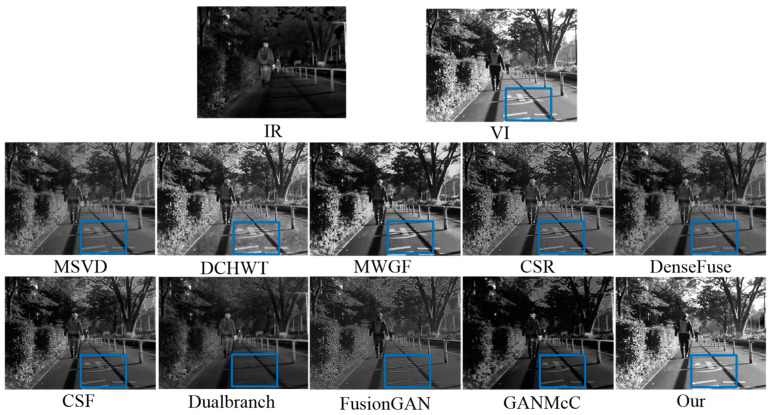
Intuitive analysis of our method and nine leading methods on the daytime images of the MSRS dataset.

The source images on the MSRS dataset contained diverse scenarios and illumination variations. We implemented testing experiments on 20 daytime infrared and visible image pairs from the MSRS dataset. Figure 11 shows one of the fusion results for the different methods. Some discriminative regions are highlighted by blue rectangles. Infrared images highlight the thermal targets, while visible images provide rich details and strong contrast and illumination. One can see that the fusion results for MSVD, CSR, DenseFuse, CSF, and GANMcC exhibited low contrast and lighting. To make matters worse, the Dualbranch and FusionGAN methods severely lost the texture details contained in the visible images, such as the words on the ground. Although the DCHWT and MWGF methods achieved relatively good fusion results, our fused image contained more and richer details and were better and brighter than theirs. These advantages can be attributed to the multi-scale feature extraction and attention-based salient feature fusion strategies included in the proposed method.

**Figure 12:**
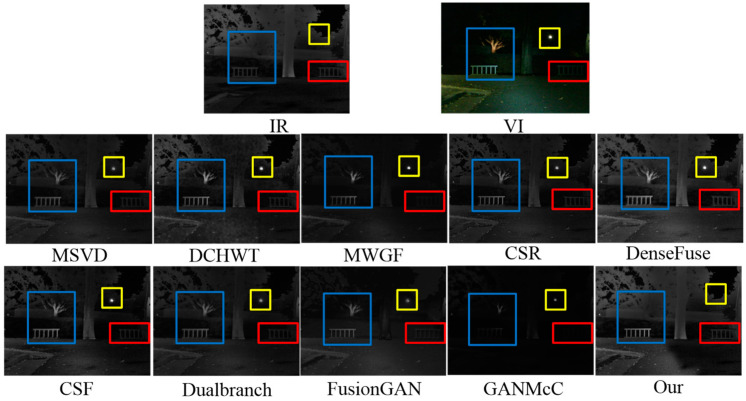
Intuitive analysis of our method and nine leading methods on the nighttime images of the MSRS dataset.

Additionally, 20 pairs of nighttime infrared and visible images from the MSRS dataset were selected for evaluation testing. In the nighttime scenario, the quality of the visible images was degraded by insufficient illumination. Hence, the fused images should have retained more texture details of the infrared images to enhance the description of the night scene. Figure 12 shows representative fusion results for the proposed method and its competitors. The discriminative regions are marked with different color boxes. Obviously, the MWGF, FusionGAN, and GANMcC methods failed to highlight the objects labeled by the red boxes. The fusion results generated by the MSVD, MWGF, CSR, DenseFuse, CSF, and Dualbranch methods were blurred in some regions. Although our method preserved more texture details from the source images, some important targets were still lost. Existing traditional and learning-based methods are designed for the fusion of infrared and visible images with normal exposure, and they do not specifically study the illumination imbalance problem. As a result, the state-of-the-art algorithms, including ours, failed to achieve satisfactory fusion results in the nighttime image fusion task. Therefore, designing robust image fusion methods that can sense illumination conditions will be a hot research topic in the future.

#### 4.5.2. Quantitative Analysis

Table 4 shows the results of the quantitative evaluation of 20 daytime image pairs on the MSRS dataset. Our metric ranked first in terms of the EN, MI, MSSIM, SF, and VIF metrics, and second in the SD metric. The best scores on the EN and MI metrics demonstrated that our fusion results contained the most information compared to other baseline methods. The largest MSSIM score indicated that our method produced fused images with minimal distortion and preserved the integrity of structural information. Leading scores on the SF and VIF metrics showed that our fused images contained richer edge and texture information and the best human visual perception. Our method achieved impressive performance on the SD metric, second only to Dualbranch. For the CC metric, the correlation was lower due to the salient feature selection operation, which reduced the linear correlation between the fused image and the two source images. For the SCD metric, our method maintained brightness and contrast close to those of the visible images, reducing the similarity between the fused image and the differential images. On the whole, our method achieved the excellent performance on all metrics.

Similarly, we also calculated the objective evaluation values for 20 pairs of nighttime infrared and visible images on the MSRS dataset. The evaluation results are shown in Table 5. Clearly, our metric was best only on the EN, SF and SD metrics. Although other comparison algorithms outperformed ours in the remaining metrics, they were still far from the best results of existing methods. In other words, the proposed method and its competitors failed to achieve excellent performance in the nighttime infrared and visible image fusion task. This is because existing traditional and learning-based fusion methods are designed for infrared and visible images with normal exposure, without considering illumination variations.

### 4.6. Ablation Experiments

We further performed ablation experiments on the TNO dataset to illustrate the necessity of the multi-scale feature extraction (MFE) and joint attention fusion (JAF) networks. The details are described below.

#### 4.6.1. Qualitative Analysis of Ablation Results

**Figure 13:**
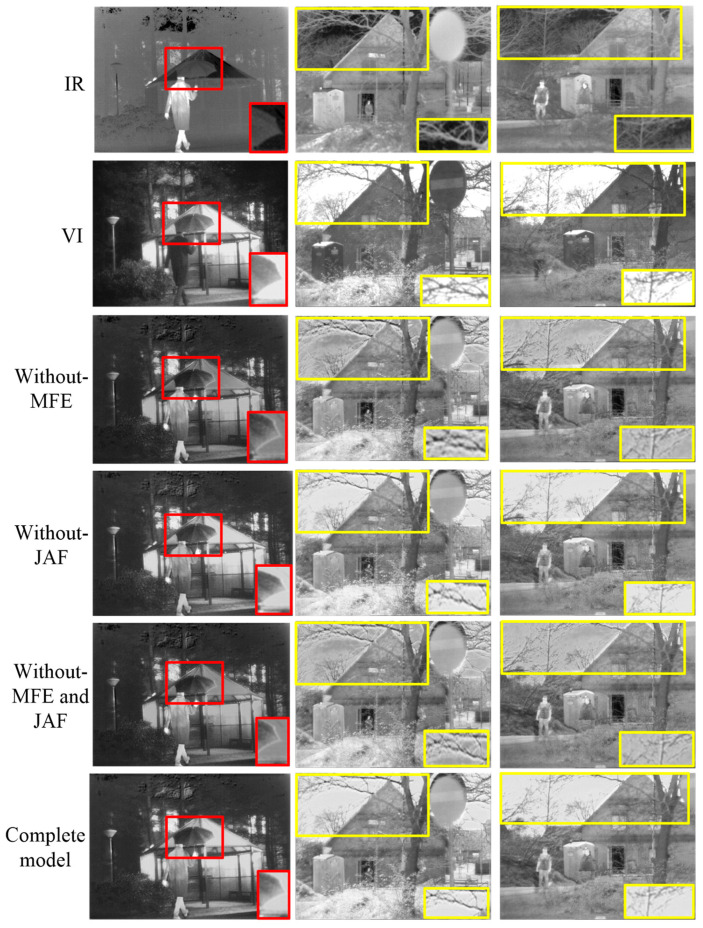
Ablation analysis of our method on the TNO dataset.

At first, the related ablation experiments were conducted using qualitative and quantitative approaches to validate the effect of the proposed multi-scale feature extraction (MFE) network. Specifically, a model termed “without-MFE” was retrained on the TNO dataset, and the others were retained. The third row of Figure 13 shows the ablation results. We could see that unnatural background texture details, such as the tree branches in the second and third columns, appeared in the fused images compared to the fused results produced by our complete model. Benefiting from the designed MFE module, our method could extract more comprehensive, deep features from the source images for fused image reconstruction.

Subsequently, the importance of the joint attention fusion (JAF) network was also demonstrated qualitatively and quantitatively. Instead of the JAF, we trained a model that obtained the fused features in a concatenation manner (termed as “without-JAF”) and compared their fusion performance. The fourth row of Figure 13 displays the ablation results. In this case, while the unnatural effects in the background were mitigated, a halo effect along the edges was introduced, such as along the edges of the umbrella. This is due to the fact that the extracted deep multi-scale features contained a large amount of redundancy and noise, which inevitably introduces artifacts into the fusion results if used directly for fused image reconstruction.

Finally, the impact of both the MFE and JAF networks on the fusion results was also tested. We removed the MFE and JAF structures simultaneously (termed as “without-MFE and JAF”) and kept the others the same as above. The fifth row in Figure 13 exhibits the corresponding ablation results. It was obvious that when the two structures (i.e., MFE and JAF) were removed, the fused images suffered from both defects of unnatural background and halo effects along the edges at the same time. This is because the meaningful information in the source images was not fully exploited in the fused image generation process.

Comparing the above ablation results with ours from the viewpoint of intuitive assessment, it was found that they all achieved good fusion performance. However, intuitively, the presence of texture detail loss and artifacts in the ablation results were still observed. We attribute the dip in performance to insufficient feature extraction and fusion. The complete model could reduce the likelihood of unnecessary artifacts by combining MFE and JAF.

#### 4.6.2. Quantitative Analysis of Ablation Results

Due to imperceptible differences in the ablation results, it was necessary to analyze them from the perspective of a quantitative evaluation. Table 6 shows the objective evaluation metrics measured on 37 image pairs from the TNO dataset. Clearly, adding the MFE and JAF yielded better performance. Therefore, through joint analysis, it was concluded that more textures could be captured from the source images into the fused images only by using both the MFE and JAF modules.

### 4.7. Comparison of Time and Space Complexity

Due to traditional algorithms included in the baseline methods run on the CPU, we only compared the time and space complexity among the various deep learning-based algorithms in Table 7. First, we computed the mean and standard deviation of the running times of different methods on the TNO, OSU, RoadScene, and MSRS datasets. Second, we counted the number of parameters of the different deep learning methods. One can see that FusionGAN achieved the minimum running time, while DenseFuse contained the smallest number of parameters. This is because FusionGAN and DenseFuse constructed the simplest structures in the testing phase. Our model was very time-consuming due to the large number of multi-scale representations and attention calculations.

## 5. Conclusions

We designed a GAN-based end-to-end method with multi-scale feature extraction (MFE) and joint attention fusion (JAF) networks (named as MJ-GAN) together with two specific, stronger discriminators that can achieve more promising fusion performance in IVIF tasks. The inventiveness of our method is that the generator implements feature extraction at different scales and utilizes the attention mechanism (AM) to fuse features in a salient way. Therefore, the difficulties of heuristic design faced by combinatorial-based and conventionally based fusion algorithms can be surmounted. Furthermore, the dual discriminator with strong discriminative ability adds more information to the fused image based on the adversarial relationships between two kinds of nets. Importantly, a hybrid loss function will guide the fusion direction and the preservation of information types from the source inputs in the final fused image. As a result, extensive experiments demonstrated the superiority of our proposed method over other representative and state-of-the-art algorithms in terms of both subjective visual quality and objective evaluation metrics.

Although the proposed image fusion method achieves competitive performance in infrared and visible image fusion tasks, there are still several issues that deserve to be highlighted. First, the proposed method is mainly aimed at grayscale image fusion, and its practical applications are limited. Second, the designed loss function only focuses on retention of the primary and secondary information of the source image, but neglects the improvement in the visual perception quality of the fused image. Third, there is still room for improvement in extracting and fusing useful features from source images. Therefore, in the future, we will try to extend the application fields and conditions for our method, such as nighttime infrared and visible image fusion, multi-focus image fusion, and multi-exposure image fusion.

## Figures and Tables

**Table 1:** The averages for the eight metrics among all methods on the TNO dataset. Bold red and bold blue represent the optimal and suboptimal results, respectively.

Methods	EN	MI	MSSIM	SF	SD	VIF	CC	SCD
MSVD	6.2910	1.5459	0.8691	9.0863	23.1439	0.3072	**0.7845**	1.5076
DCHWT	6.3624	1.4758	0.8591	9.1122	25.3286	0.3170	0.7609	1.4793
MWGF	**6.8143**	1.9285	0.6770	**10.9906**	31.8245	0.1677	0.7089	0.9596
CSR	6.3558	1.5582	**0.8919**	9.5963	24.3851	0.3261	0.7771	1.5299
DenseFuse	6.7256	1.7079	0.8445	10.0688	**32.8838**	**0.5303**	0.7767	**1.5671**
CSF	6.2718	1.4957	0.8215	6.6046	23.3368	0.2831	0.7653	1.4056
Dualbranch	6.6357	1.7618	0.8364	8.8754	28.5149	0.3290	0.7665	1.5054
FusionGAN	6.6357	1.7618	0.8364	8.8754	28.5149	0.3290	0.7665	1.5054
GANMcC	6.4598	**2.1528**	0.8544	7.6292	29.6072	0.3520	0.7306	1.1864
Ours	**6.8979**	**2.0605**	**0.9102**	**10.0811**	**39.3882**	**0.5333**	**0.7857**	**1.6021**

**Table 2:** The averages for the eight metrics among all methods on the OSU dataset. Bold red, bold blue and bold green represent the best, second-best and third-best results, respectively.

Methods	EN	MI	MSSIM	SF	SD	VIF	CC	SCD
MSVD	7.2833	2.6055	0.8521	25.3853	43.1537	0.3705	**0.8441**	1.2627
DCHWT	**7.5213**	2.5685	0.8142	**28.824**	**49.4463**	0.3472	0.8341	1.2625
MWGF	**7.6195**	2.7098	0.6600	26.9111	**51.7509**	0.2449	0.8192	0.8987
CSR	7.3895	2.6396	**0.8760**	**28.4073**	46.1330	0.3821	0.8436	1.2927
DenseFuse	7.2631	**3.0613**	**0.8619**	18.6400	42.9906	**0.3998**	**0.8491**	**1.3160**
CSF	7.4299	2.7373	0.8515	18.7358	47.8021	**0.4110**	0.8417	**1.3636**
Dualbranch	7.2669	**2.9374**	0.8043	25.9692	45.2490	0.3296	0.8291	1.0696
FusionGAN	7.2844	2.3880	0.8165	25.0017	44.8362	0.3616	0.8281	1.1857
GANMcC	7.1948	2.7391	0.8182	19.0651	44.2739	0.3642	0.8386	1.2058
Ours	**7.5510**	**3.0675**	**0.8592**	**30.3577**	**64.7876**	**0.4357**	**0.8494**	**1.4693**

**Table 3:** The averages for the eight metrics among all methods on the RoadScene dataset. Bold red, bold blue and bold green represent the best, second-best and third-best results, respectively.

Method	EN	MI	MSSIM	SF	SD	VIF	CC	SCD
MSVD	6.8385	2.6733	0.8596	11.2012	31.5209	0.3499	**0.7904**	1.2882
DCHWT	7.2092	2.7422	0.8687	11.9652	**65.6307**	0.4187	0.7713	1.2821
MWGF	7.2488	2.7267	0.8410	10.4601	**47.9735**	0.3888	0.7385	0.9468
CSR	6.9308	2.7179	**0.9090**	**12.4601**	33.2636	0.4069	**0.7892**	1.3046
DenseFuse	7.1912	2.9594	0.8278	11.1098	41.7094	**0.5403**	0.7869	1.2294
CSF	**7.3976**	2.8815	**0.9320**	12.3282	46.2293	**0.5920**	0.7890	1.2318
Dualbranch	7.0685	**2.9691**	0.7584	**13.1972**	36.8233	0.3051	0.7600	1.1011
FusionGAN	**7.3255**	**2.9797**	0.8358	8.5244	47.7102	0.4102	0.7672	**1.4583**
GANMcC	7.2460	**3.2011**	0.8841	10.3838	46.3536	0.4661	0.7581	**1.3714**
Ours	**7.5183**	2.8357	**0.8988**	**14.0514**	**51.7240**	**0.5447**	**0.8124**	**1.6672**

**Table 4:** The averages for the eight metrics among all methods on the daytime images of the MSRS dataset. Bold represents the optimal results.

Method	EN	MI	MSSIM	SF	SD	VIF	CC	SCD
MSVD	6.6021	2.8084	0.8697	10.5125	30.1600	0.3656	0.7700	1.5829
DCHWT	7.2387	2.3056	0.8416	13.1350	44.3694	0.5979	0.7381	1.5219
MWGF	7.0129	2.5484	0.9351	12.3888	53.6275	0.6959	0.7205	1.2018
CSR	6.7244	2.7870	0.9312	12.2905	32.5781	0.4802	0.7651	1.5708
DenseFuse	6.8581	3.1902	0.9250	9.6056	37.3920	0.5286	0.7772	**1.6750**
CSF	6.6866	2.6980	0.8828	9.4354	33.5528	0.4443	0.7948	1.6062
Dualbranch	6.5850	1.9526	0.3717	13.7977	**63.9720**	0.1377	0.6280	0.6872
FusionGAN	5.9921	2.4996	0.7581	10.3930	19.2707	0.1804	**0.7954**	1.1804
GANMcC	5.8653	2.7971	0.7571	6.5153	27.3598	0.2820	0.7635	1.4200
Ours	**7.4177**	**3.5244**	**0.9364**	**14.7850**	56.4928	**0.6997**	0.7496	1.5244

**Table 5:** The averages for the eight metrics among all methods on the nighttime images of the MSRS dataset. Bold represents the optimal results.

Method	EN	MI	MSSIM	SF	SD	VIF	CC	SCD
MSVD	5.5665	2.2846	0.9235	6.1290	20.6210	0.4083	0.7505	1.6688
DCHWT	5.8037	2.0093	0.9395	7.3825	28.4595	**0.6260**	0.7290	1.6166
MWGF	4.9787	2.6874	0.9074	7.4880	26.8118	0.5597	0.6848	1.3698
CSR	5.6038	2.2521	**0.9469**	6.7443	22.1749	0.5131	0.7455	1.6663
DenseFuse	5.7305	2.5001	0.9398	5.2297	23.4307	0.5311	0.7488	**1.7466**
CSF	5.2898	2.3370	0.9145	4.9344	20.9921	0.4550	0.7409	1.6492
Dualbranch	5.5508	2.4437	0.9140	5.0897	20.1504	0.3949	**0.7539**	1.6549
FusionGAN	4.9961	**3.0527**	0.8061	4.9658	14.6956	0.2033	0.6699	0.7486
GANMcC	3.3486	1.7811	0.7790	3.4595	14.1554	0.2040	0.6422	1.2506
Ours	**5.8273**	2.3644	0.8816	**7.5758**	**33.8715**	0.4742	0.6721	1.3531

**Table 6:** The averages for the six metrics among all models on the TNO dataset. Bold represents the optimal results.

Methods	MI	MSSIM	SF	SD	CC	SCD
Without-MFE	1.6808	0.8974	8.9034	37.0276	0.7411	1.5119
Without-JAF	1.6780	0.8982	8.2670	37.9932	0.7477	1.5280
Without-MFE and JAF	1.6981	0.89178	8.5157	36.2963	0.7212	1.5343
Complete model	**2.0605**	**0.9102**	**10.0811**	**39.3882**	**0.7845**	**1.6021**

**Table 7:** Time and space complexity of different image fusion methods.

Items		DenseFuse	CSF	Dualbranch	Fusion-GAN	GANMcC	Ours
Run time/s	TNO	0.77±0.90	5.04±2.17	1.04±0.07	0.12±0.60	0.28±0.77	0.26±0.78
	OSU	0.96±1.10	4.77±2.11	1.69±0.17	0.15±0.66	0.18±0.74	0.23±0.97
	Road-Scene	2.84±1.05	10.45±2.87	3.39±0.52	0.85±0.66	1.06±0.73	1.34±0.92
	MSRS	1.02±1.32	11.09±4.31	6.35±0.60	0.24±0.81	0.33±1.04	0.34±1.14
parameters/K		73.4	185.4	89.5	925.6	186.7	302.4

## Data Availability

The data presented in this study are available on request from the corresponding author. The data is not publicly available because the code of this manuscript involves future research that has not yet been done by the authors.
